# Test-Enhanced E-Learning Strategies in Postgraduate Medical Education: A Randomized Cohort Study

**DOI:** 10.2196/jmir.6199

**Published:** 2016-11-21

**Authors:** Lisa A DelSignore, Traci A Wolbrink, David Zurakowski, Jeffrey P Burns

**Affiliations:** ^1^ Division of Critical Care Medicine Department of Anesthesia, Perioperative, and Pain Medicine Boston Children's Hospital Boston, MA United States; ^2^ Department of Anesthesia, Perioperative, and Pain Medicine Boston Children's Hospital Boston, MA United States; ^3^ Department of Anesthesia Harvard Medical School Boston, MA United States

**Keywords:** distance learning, computer-assisted instruction, medical education, educational measurement, retention

## Abstract

**Background:**

The optimal design of pedagogical strategies for e-learning in graduate and postgraduate medical education remains to be determined. Video-based e-learning use is increasing, with initial research suggesting that taking short breaks while watching videos (independent of answering test questions) may improve learning by focusing attention on the content presented. Interspersed test questions may also improve knowledge acquisition and retention.

**Objective:**

To examine the effect of interspersed test questions and periodic breaks on immediate knowledge acquisition and retention at 6 months by pediatric residents engaged in video-based e-learning.

**Methods:**

First- and second-year pediatric residents were randomized to 1 of the following 3 groups: viewing the complete video uninterrupted (full video), viewing the video interrupted with unrelated logic puzzles (logic puzzles), or viewing the video interrupted with brief comprehension test questions (short answer questions). Residents answered pre- and post-tests before and after video viewing, followed by a retention test at 6 months. Primary outcome included comparison of the change in test scores between groups.

**Results:**

A total of 49 residents completed the initial testing session. All 3 learning groups had comparable mean increases in immediate knowledge gain, but with no significant differences between groups (*F*_2,46_=0.35, *P*=.71). Thirty-five residents completed retention testing with comparable degrees of knowledge retention in the full video and short answer test questions groups (*P*<.001), but no significant change in the logic puzzles group (*F*_1,32_=2.44, *P*=.13).

**Conclusions:**

Improved knowledge gain was not demonstrated among residents answering interspersed questions or completing logic puzzles during interrupted online video viewing when compared with residents viewing uninterrupted video content. However, residents who either participated in uninterrupted video viewing or answered interspersed questions during interrupted video viewing demonstrated significant knowledge retention at 6 months.

## Introduction

The recent introduction of e-learning initiatives in postgraduate medical education has been heralded as a disruptive change to efficiently scale knowledge and promote more effective learning. Although e-learning can deliver knowledge, research to inform the optimal learning design and teaching practices in this environment remains in its infancy [1-4]. Research in the cognitive psychology literature demonstrates that test-enhanced learning, that is, answering test questions at repeated intervals during an educational activity, improves knowledge gain in both classroom [5,6] and e-learning settings by encouraging active information retrieval, focusing attention on the content presented, promoting task-relevant behaviors such as note-taking, and reducing overall cognitive demand [7].

Using interspersed test questions as an educational learning tool also allows for superior knowledge retention relative to passively restudying the same material when students are tested at extended intervals after the initial learning activity [8,9]. Tests that stimulate deeper retrieval of information, such as short answer or essay, have the potential to achieve better knowledge gains than recall tests such as simple multiple choice questions [10,11]. Multiple choice questions can further be classified as those that require clinical knowledge application, or context-rich questions, versus those that require simple factual recall, or context-free questions [11].

Despite the increased use of e-learning platforms to educate postgraduate medical trainees, we were unable to identify any studies investigating the implementation of test-enhanced learning strategies in an e-learning platform for postgraduate medical education. Yet, many e-learning platforms and massive open online courses (MOOCs) utilize uninterrupted instructional videos as a means of learning, with or without pre- and post-tests for knowledge assessment [12-14].

The purpose of this study was to evaluate the extent to which the use of interspersed test questions or taking periodic breaks while watching an online video would impact knowledge gain as compared with watching the same video without any breaks. As a proxy for knowledge gain, the primary study outcome was the difference between pre- and post-test scores between groups. Secondary outcomes included the difference between pre- and post-test scores within each group and the difference in retention test scores at 6 months compared with pretest scores between groups.

## Methods

### Recruitment and Study Design

We conducted a randomized, prospective, cohort study in 3 academic medical centers in Boston, Massachusetts between June 2014 and March 2016. Pediatric residents in their first or second year of postgraduate training were eligible to participate. Participation was completely voluntary, as this educational initiative was independent from educational obligations during their clinical pediatric intensive care unit (PICU) rotations. Residents were ineligible if they had previously completed a 4-week PICU rotation during residency because the study intervention assessed knowledge acquisition and retention of mechanical ventilation concepts that would have been encountered by residents on their first PICU rotation. Email invitations were sent to all eligible residents enrolled in the following pediatric residency programs: Boston Combined Pediatric Residency Program, Massachusetts General Hospital, and Tufts Floating Hospital for Children. Residents provided voluntary written consent for study inclusion. No residents who volunteered to participate actively refused participation at a subsequent time point during the study. Residents received a US $50 Amazon gift card and the chance to receive an iPad via random selection upon completion of 6-month retention testing. The Boston Children’s Hospital’s Institutional Review Board deemed this study exempt from informed consent, given no identifying data on study participants were collected. Affiliated institutions honored the exempt status. Initial testing was conducted at each of the 3 institutions with all sessions monitored by 1 of 2 study facilitators who were available to troubleshoot technical difficulties and monitor for dishonest behavior. Retention tests were administered via email. Residents were asked to abide by the honor code when completing the retention test.

**Figure 1:**
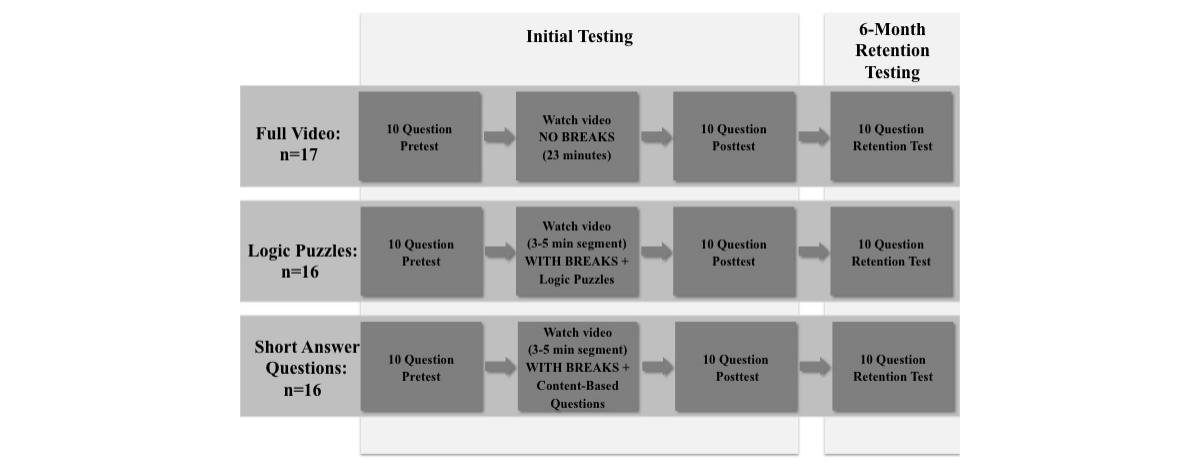
Schematic design of randomized study protocol for the 3 learning groups.

Pediatric residents were blindly randomized via concealed envelopes to 1 of the following 3 groups: full video, logic puzzles, or short answer questions (Figure 1). Residents in the full video group watched the video uninterrupted (without breaks), representing the “control” group as this is the typical e-learning video format. Residents in the logic puzzles and short answer questions groups watched the same video with interspersed breaks during which they either completed noncontextual logic puzzles or content-based test questions.

### Study Materials

Residents completed all computer-based elements of this study via a single lesson plan created on the commercial e-learning platform Softchalk (Softchalk LLC, Richmond, VA). All residents watched a peer-reviewed video about the basic principles of high frequency oscillatory ventilation (HFOV), assuming that pediatric residents would have limited baseline knowledge of this content as clinical exposure to ICUs is low early in residency training. In addition, this specific topic was chosen for educational use because although HFOV is an important mode of mechanical ventilation for advanced, refractory pediatric respiratory failure and requires basic conceptual understanding by residents, the use of this type of mechanical ventilation is an overall low-frequency event in most PICUs. The video was written and presented by a Harvard Professor who conducts research on HFOV, as part of an existing curriculum on OPENPediatrics, an open access, e-learning platform [12]. This video was peer-reviewed by mechanical ventilation content experts. OPENPediatrics has been integrated into the Boston Combined Residency Program curriculum such that residents rotating through the PICU must complete video-based lessons on OPENPediatrics, including those related to HFOV. Thus, survey data collected the information whether the residents had ever logged into OPENPediatrics and watched HFOV-related videos. OPENPediatrics verified whether study participants viewed the HFOV video on OPENPediatrics during the study timeline.

Three content experts from the Division of Critical Care at Boston Children’s Hospital developed test questions and acceptable answers. All experts utilized the same content validity scoring system to evaluate questions. Questions scored as highly relevant (ie, score of 3 or 4) were included. All questions required free text answers. During the initial testing session, all residents completed a 10-question pretest prior to video watching and a 10-question posttest immediately afterwards. Six months after initial testing session completion, all residents were asked to complete a 10-question retention test. Different questions were included on pre-, post-, and retention tests, but all tested similar concepts. Three independent graders scored all test questions upon participant completion. Free text responses were scored on a binary scale (0 points=incorrect, 1 point=correct). Scores were reported as percent correct, out of a possible 100% (ie, 10 points out of 10 questions=100%). No partial credit was given.

### Statistical Analysis

A biostatistician with several decades of experience in the field reviewed and approved the analytic plan used to evaluate the data. Pearson chi-square tests and one-way analysis of variance (ANOVA) were used to compare baseline demographic characteristics between groups. One-way ANOVA was used with *F*-tests to compare differences in the change in pre- and post-test scores among the 3 groups immediately and at 6-month follow-up [15]. Repeated measures mixed-model ANOVA was used to compare the changes in test scores within each group [15]. Statistical analysis reported results as mean percent correct test scores with associated 95% CIs. ANOVA analyses were performed using SPSS statistical software version 23.0 (IBM Corporation). Two-tailed values of *P*<.05 were considered statistically significant.

### Sample Size Calculations

Power calculations indicated that 16 residents randomized to each of the 3 learning groups would provide 80% statistical power (two-tailed alpha=.05, beta=.20) to detect a 20% mean difference at immediate posttest evaluation and 6-month retention test evaluation, assuming a pooled standard deviation of 18-20% (approximate effect size=1.1) (version 7.0, nQuery Advisors, Statistical Solutions, Cork, Ireland).

## Results

### Descriptive Characteristics

A total of 49 pediatric residents completed the initial testing session. Table 1 reports the baseline characteristics of each group prior to initial pretest. The majority of residents in all groups had little exposure to ICU rotations, both as residents and medical students. Residents self-reported a wide exposure range in caring for ventilated patients with 94% (46/49) reporting limited exposure in caring for patients ventilated by HFOV (<5 patients). Residents in all groups reported no prior exposure to HFOV-related video content on OPENPediatrics. Review of each participant’s video viewing activity within OPENPediatrics verified residents’ self-reported lack of prior video content exposure.

**Table 1:** Residents’ baseline demographic characteristics (overall and by group). Previous ICU experiences represent each individual’s combined experiences as a medical student and resident.

Baseline characteristics			All groups (N=49)	Full video (n=17)	Logic puzzles (n=16)	Short answer questions (n=16)	*P* value
Age in years (Mean)			25-36 (28)	25-32 (27.6)	25-35 (27.8)	25-36 (29.6)	.57
	**Gender, n (%)**						.19
		Male	17 (35%)	3 (18%)	7 (44%)	7 (44%)	
		Female	32 (65%)	14 (82%)	9 (56%)	9 (56%)	
	**Degree, n (%)**						.31
		MD	41 (84%)	15 (88%)	15 (94%)	11 (69%)	
		MD-PhD	7 (14%)	2 (12%)	1 (6%)	4 (25%)	
		Other	1 (2%)	-	-	1 (6%)	
	**Field of residency training, n (%)**						.38
		Pediatrics	41 (84%)	16 (94%)	14 (88%)	11 (69%)	
		Internal medicine-pediatrics	4 (8%)	-	1 (6%)	3 (19%)	
		Combined pediatrics-neurology	2 (4%)	1 (6%)	-	1 (6%)	
		Combined pediatrics-anesthesia	2 (4%)	-	1 (6%)	1 (6%)	
	**Current year of residency training, n (%)**						.99
		PGY^a^-1	31 (63%)	11 (65%)	10 (63%)	10 (63%)	
		PGY-2	18 (37%)	6 (35%)	6 (37%)	6 (37%)	
**Combined previous intensive care unit experience**							
	**PICU^b^, n (%)**						.89
		0 Months	33 (67%)	13 (76%)	10 (63%)	10 (63%)	
		1 Month	13 (27%)	3 (18%)	5 (31%)	5 (31%)	
		≥2 Months	3 (6%)	1 (6%)	1 (6%)	1 (6%)	
	**NICU^c^, n (%)**						.44
		0 Months	12 (24%)	3 (18%)	3 (19%)	6 (38%)	
		1 Month	13 (27%)	5 (29%)	6 (37%)	2 (12%)	
		≥2 Months	24 (49%)	9 (53%)	7 (44%)	8 (50%)	
	**MICU^d^ (adult), n (%)**						.08
		0 Months	41 (84%)	17 (100%)	12 (75%)	12 (75%)	
		1 Month	8 (16%)	-	4 (25%)	4 (25%)	
	**SICU^e^ (adult), n (%)**						.60
		0 Months	47 (96%)	16 (94%)	16 (100%)	15 (94%)	
		1 Month	2 (4%)	-	-	2 (12%)	
	**CICU^f^ (adult), n (%)**						.12
		0 Months	47 (96%)	17 (100%)	16 (100%)	15 (94%)	
		≥2 Months	2 (4%)	-	2 (12%)	-	
	**Burn ICU, n (%)**						.12
		0 Months	47 (96%)	17 (100%)	14 (88%)	16 (100%)	
		1 Month	2 (4%)	-	2 (12%)	-	
**Previous experience in care of ventilated patients**							
	**Conventional mechanical ventilation, n (%)**						.62
		0-5 patients	13 (27%)	4 (23%)	5 (31%)	4 (25%)	
		6-10 patients	16 (33%)	8 (47%)	5 (31%)	3 (19%)	
		11-15 patients	11 (22%)	2 (12%)	4 (25%)	5 (31%)	
		>16 patients	9 (18%)	3 (18%)	2 (13%)	4 (25%)	
	**High frequency oscillatory ventilation, n (%)**						.72
		0-2 patients	33 (67%)	11 (65%)	11 (69%)	11 (69%)	
		3-5 patients	13 (27%)	4 (23%)	5 (31%)	4 (25%)	
		6-8 patients	3 (6%)	2 (12%)	-	1 (6%)	
**Previous experience with OPENPediatrics**							
	**Personal log-in attempts, n (%)**						.99
		0	33 (68%)	12 (70%)	11 (69%)	10 (63%)	
		1	5 (10%)	1 (6%)	2 (12%)	2 (12%)	
		2	6 (12%)	2 (12%)	2 (12%)	2 (12%)	
		≥3	5 (10%)	2 (12%)	1 (6%)	2 (12%)	

^a^PGY: postgraduate year.

^b^PICU: pediatric intensive care unit.

^c^NICU: neonatal intensive care unit.

^d^MICU: medical intensive care unit.

^e^SICU: adult surgical intensive care unit.

^f^CICU: cardiac intensive care unit.

### Initial Testing Session Analysis

**Figure 2:**
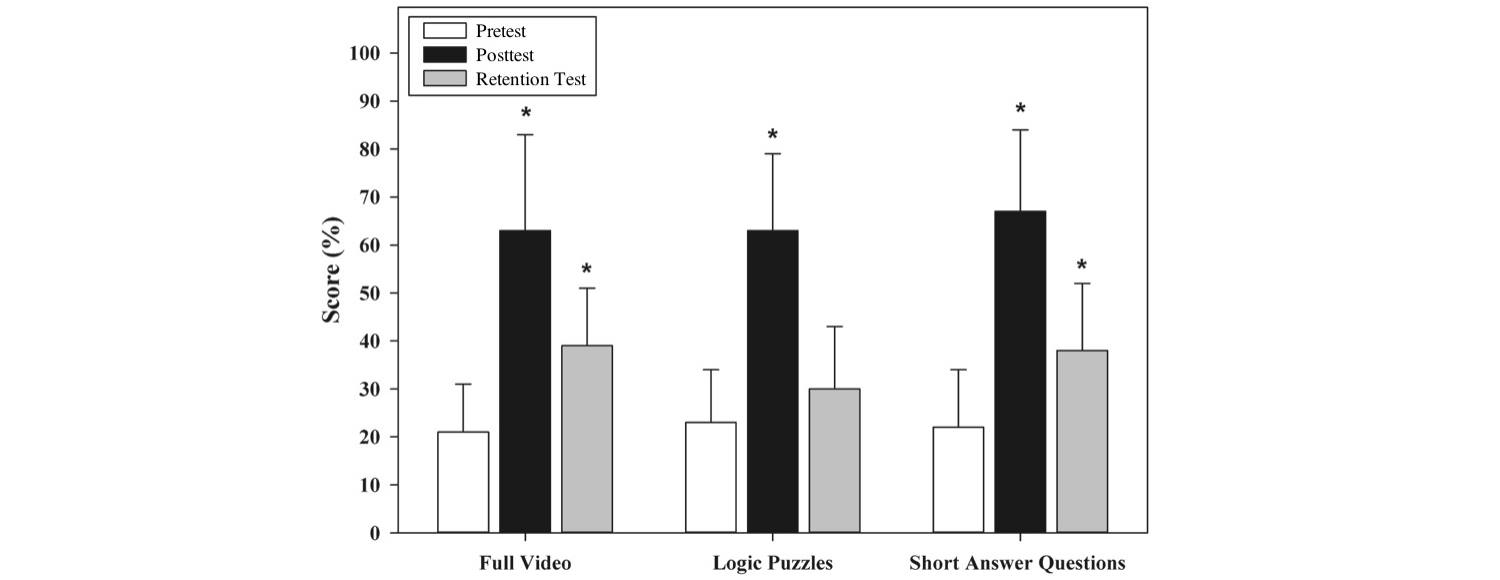
Residents’ mean percent correct pretest, posttest, and 6-month retention scores according to group. Error bars represent standard deviation in each group. Asterisks represent statistical significance (*P*<.001) in scores when compared with pretest scores.

Pediatric residents were randomized to the following groups: full video (n=17), logic puzzles (n=16), and short answer questions (n=16). Mean initial pre- and post-test percent correct scores and 95% CIs for each group are reported in Tables 2 and 3 and represented in Figure 2. Mixed-model ANOVA showed significant improvement in knowledge gain between pre- and post-test scores in each of the 3 groups during the initial testing session (full video: *F*_1,46_=80.52, *P*<.001; logic puzzle: *F*_1,46_=67.36, *P*<.001; short answer questions: *F*_1,46_=87.98, *P*<.001). One-way ANOVA revealed comparable mean improvement in the change in the test score from pre- to post-test in all 3 groups during the initial testing session (*F*_2,46_=0.35, *P*=.71). Adjustment for gender and postgraduate training year did not alter the overall results, although second-year residents randomized to the short answer questions group had greater percent improvement in posttest scores compared with first-year residents (63% [SD 13] vs 39% [SD 16] vs *P*=.02).

**Table 2:** Residents’ mean percent correct test scores by group for initial testing. Mean difference in test score at 6-months follow-up represents the difference between initial pretest and 6-month follow-up test.

Group	Mean percent correct pretest score % (95% CI)	Mean percent correct posttest score % (95% CI)	Mean difference in percent correct test score % (95% CI)	*P* value
Full video (n=17)	21 (14-28)	63 (56-70)	42 (32-52)	<.001
Logic puzzle (n=16)	23 (16-31)	63 (55-70)	39 (29-50)	<.001
Short answer questions (n=16)	22 (14-29)	67 (60-74)	45 (35-55)	<.001

**Table 3:** Residents’ mean percent correct test scores by group for 6-month retention testing. Mean difference in test score at 6-months follow-up represents the difference between initial pretest and 6-month follow-up test.

Group	Mean percent correct retention test score % (95% CI)	Mean difference in percent correct test score % (95% CI)	*P* value
Full video (n=12)	39 (31-47)	18 (8-28)	<.001
Logic puzzle (n=11)	30 (21-38)	7 (-3-17)	<.001
Short answer questions (n=11)	39 (31-47)	17 (7-27)	<.001

### Six-Month Retention Test Analysis

Thirty-five residents (71%; 35/49) completed the 6-month retention test with similar numbers in each group (full video: n=12; logic puzzles: n=11; short answer questions: n=12). During this 6-month interval, 10 residents overall rotated through the PICU (full video: n=5; logic puzzles: n=3; short answer questions: n=2; *P*=.80), all reporting caring for at least 3 patients receiving HFOV. No residents cared for more than 5 patients receiving HFOV (*P*=.67). Five residents reported repeated viewing of the HFOV video on OPENPediatrics prior to the 6-month posttest (full video: n=1, logic puzzles: n=3, short answer questions: n=1). Cross-reference of OPENPediatrics data verified that only 2 participants had actually watched the video in between the initial and 6-month retention testing. Mean retention test scores for each group are reported in 3.

One-way ANOVA revealed that all 3 groups had comparable degrees of mean knowledge retention between the initial pretest and retention test (*F*_2,32_=2.77, *P*=.08). Repeated measures mixed-model ANOVA demonstrated that the full video and short answer questions groups had a significant degree of knowledge retention at 6 months, although to a lesser degree than knowledge gained on initial mean posttest scores (full video: *F*_1,32_=22.08, *P*<.001; short answer questions: *F*_1,32_=20.12, *P*<.001). The logic puzzle group did not demonstrate statistically significant knowledge retention at 6 months (*F*_1,32_=2.44, *P*=.13).

## Discussion

### Principal Findings

This study did not demonstrate a significant difference related to overall initial knowledge gain when pediatric residents watched a video without structured breaks compared with those with interspersed breaks and completion of either logic puzzles or short answer questions. Despite this finding, we demonstrate that irrespective of group assignment, all groups had significantly higher mean percent correct test scores on initial posttests compared with pretests. This suggests that the online video itself is an effective teaching modality. Six-month retention analysis reveals a continued lack of statistical significance when comparing residents’ change in test scores between groups. However, there is an overall significant increase in the change in test scores between initial pretests and 6-month retention tests in the full video and short answer groups (*P*<.001), although to a lesser degree compared with the change in test scores between initial pre- and post-testing.

Although not statistically significant, the short answer questions group’s mean change in test score demonstrates the largest percentage point difference in comparison of pre- to post-test score, consistent with previous literature supporting improved knowledge gain via the use of interspersed short answer test questions [3,5-11]. It is not surprising that our 6-month retention analysis demonstrates a lesser degree of knowledge gain compared with initial pre- and post-testing within each group, especially as there were no additional testing intervals prior to the 6-month retention test. This is consistent with the cognitive psychology theory of spaced learning, suggesting the need for more frequent testing intervals to improve ongoing knowledge retention at 6 months [16-21]. What remains curious is why pediatric residents in the logic puzzles group did not retain as significant knowledge between the initial pretest and retention test as the other 2 groups, despite demonstrating a knowledge gain between initial pre- and post-testing. This is most likely attributable to small sample size, but raises the question of whether this type of mind-engagement, although active, negatively affects long-term knowledge transfer by increasing cognitive load.

### Comparison With Prior Work

We are unaware of any prior studies similar to our study design and findings; however, in recent years, test-enhanced learning has been studied in various online educational settings [3,5,10,22]. Szpunar et al (2013) studied undergraduates taking an online statistics course and found that interspersing test questions while watching an online lecture not only improved overall learning, but also encouraged task-relevant note-taking activities and discouraged mind-wandering activities when compared with students passively reviewing the lecture content [3]. A few questions arise from this study including the specific timing and frequency of interspersed test questions, the type and format of questions used (content-relevant or not), and whether just taking periodic breaks with mind activation during an educational activity can improve knowledge gain. Cook et al (2014) investigated what may represent the optimal number of interspersed questions in the context of e-learning, suggesting that there may be a critical number of questions ideal for enhancing learning, above which no additional learning benefit is acquired [21]. McConnell et al (2015) demonstrated equivalence between short answer questions and context-rich multiple choice questions in mock licensure exam score improvement among Canadian medical students; yet, both of these educational strategies remained superior to restudying and context-free multiple choice questions [11]. Finally, if just taking breaks during an educational activity improves knowledge gain, then it would be important to understand how the specific activity one performs during those breaks affects knowledge gain.

### Strengths and Limitations

The strengths of this study include the overall design involving 3 independent groups with comparison of 2 active interventions, the quality of educational material used, and the high follow-up rate for 6-month retention evaluation. The exact reason for residents who were lost to follow-up at 6 months is unknown (n=15), but possibly due to time constraints related to clinical rotations or time away from residency during which they were unresponsive to email.

We acknowledge several limitations to this study. First, the sample size reported here was designed to power to an 80% level, yet it is still possible that our sample size was too small to detect significant differences between groups. Second, the lack of a statistically significant difference in knowledge gain between groups may be related to several factors, including overall video duration, timing of when residents completed this study in the context of their clinical rotations, emotional state of residents during study completion (ie, level of fatigue, anxiety, distraction), and their overall content interest. Third, this study may have some methodological insufficiency regarding the use of spaced learning for evaluation of knowledge retention, and we believe that this would be an interesting hypothesis to incorporate in a future study.

The overall duration of the video used in this study was relatively short (23 min) such that this may have contributed to not finding a meaningful effect size difference between groups who were taking breaks while watching the video. Yet, data regarding the optimal video length for learner engagement are conflicting. Research in disciplines other than medicine suggests that shorter duration is generally better and that including breaks within longer videos helps reduce cognitive load. Data from TED talks suggests that the optimal video length is around 18 min, which is short enough to hold attention, yet long enough to succinctly communicate complex topics, both of which decreases cognitive overload by limiting the amount of time of active brain engagement, and forces the speaker to be clear and concise [22]. Data from EdX blog, an open-source e-learning platform and MOOC provider, support that longer videos should be divided into smaller segments, with preliminary evidence demonstrating that for students enrolled in various math and science courses, the optimal video length for engagement was between 6 and 9 min [23]. More rigorous study of the optimal timing for video-based e-learning in the context of medical education is warranted to determine and reinforce these concepts.

In addition, inattentiveness and mind-wandering have been linked to poor knowledge gain, and these behaviors occur more frequently when students are experiencing an underlying negative emotional state, lack engagement, or experience stress related to learning [24]. These are all prominent factors encountered in postgraduate medical training, and as such may have affected some residents in this study. Moreover, residents did not receive immediate feedback after answering test questions during this study to avoid confounding 6-month retention test results by restudying material. This lack of immediate feedback could have negatively affected long-term learning in this context.

Finally, several limitations must be considered when reviewing our secondary outcome of retention test score analysis. First, residents were not directly observed for dishonest behavior on retention test completion, which could potentially falsely elevate test scores. Second, we did not specifically control for “on-the-job” training. However, given the overall small exposure to patients ventilated by HFOV as self-reported by residents across all groups between the initial testing and the 6-month follow-up, we do not believe this has greatly impacted our findings as differences in exposure between groups lacked statistical significance (*P*=.67). If this were clinically significant, we would have expected to observe a greater increase in knowledge retention within all groups at 6 months’ follow-up. Similarly, numbers of residents rotating through the PICU between initial testing and 6-month follow-up were low and not statistically different between groups (*P*=.80). We continue to acknowledge the overall small sample size in interpretation of our 6-month follow-up analysis.

### Conclusions

In summary, this cohort study of pediatric residents did not demonstrate similar findings to those reported by Szpunar (2013), in which interspersed test questions and periodic breaks integrated into an online statistics lecture improved knowledge gain among undergraduate students. However, when our findings are viewed together with other previous studies [3,7,10,21], we find there is a continued need to investigate optimal strategies for augmenting learning and retention in video-based e-learning, with ongoing consideration of the need to integrate periodic breaks, interspersed test questions, and spaced learning intervals, in addition to determining optimal video length. Future e-learning platforms will also need to support robust analytics for data collection of privacy-protected, deidentified data that will better inform research on optimal learning strategies and technologies going forward.

## Abbreviations

HFOV: High frequency oscillatory ventilation
MOOC: Massive open online courses
NICU: Neonatal intensive care unit
PICU: Pediatric intensive care unit
